# The Effect of Deproteinized Bovine Bone Mineral on Saos-2 Cell Proliferation

**Published:** 2013-08-01

**Authors:** Arash Khojasteh, Mohammad Hossein Ghahremani, Seyed Nasser Ostad, Mohammad Eslami, Pourya Motahhary, Golnaz Morad, Shireen Shidfar

**Affiliations:** a Department of Oral and Maxillofacial Surgery, Research Institute of Dental Sciences, Dental School, Shahid Beheshti University of Medical Sciences, Tehran, Iran; b Department of Pharmacology and Toxicology, Faculty of Pharmacy, Tehran University of Medical Sciences, Tehran, Iran; c Department of Oral and Maxillofacial Pathology, Tehran University of Medical Sciences, Tehran, Iran; d Dental Research Center, Research Institute of Dental Sciences, Shahid Beheshti University of Medical Sciences, Tehran, Iran; e Gifted and Talented Dental Students Division, Dental School, Shahid Beheshti University of Medical Sciences, Tehran, Iran

**Keywords:** Bovine Bone Mineral, Cell Proliferation, Dexamethasone, Osteoblast, Tissue Scaffold, Xenograft

## Abstract

**Introduction:**

Deproteinized bovine bone mineral (Bio-Oss) is a xenogenic bone substitute, widely used in maxillofacial bone regeneration. The aim of this in vitro study was to investigate its influence on the growth behavior of human osteosarcoma cell line, Saos-2 culture, and compare it with the physiologic dose of Dexamethasone, an inductive factor for osteoblasts.

**Materials and Methods:**

Human osteosarcoma cells, Saos-2, were cultured on Bio-Oss and their growth rate was compared to Saos-2 cultures treated with Dexamethasone 10^-7^ M in contrast to cells cultivated in PBS, in the control group. Assessment of proliferation was performed after 24, 36, and 48 hours by counting cells using trypan blue exclusion method. Alkaline phosphatase was measured spectrophotometrically at 405 nm with paranitrophenol buffer.

**Results:**

After 48 hours, the number of Saos-2 cells increased significantly when subcultured with Bio-Oss. Bio-Oss was more effective on the enhancement of proliferation of Saos-2 cells when compared to the physiologic dose of Dexamethasone (P<0.05). Alkaline phosphatase activity increased in cells grown on Bio-Oss and dexamethasone 10^-7^ M in contrast to cells cultivated in PBS control group. The greatest level of activity was observed in the group containing Bio-Oss after 48 hour.

**Conclusion:**

The significant increase of cell proliferation and alkaline phosphatase activity in cells cultured on Bio-Oss, compared to Dexamethasone-treated cells, suggests the important role of this bone substitute in promoting bone regeneration.

## Introduction

During the past decades, the concept of utilizing a combination of cells and signaling molecules to activate/advance the passive effects of different osteoconductive scaffolds and create an active bone regeneration approach, has become central to dental research [1].

The physical characteristics and chemical composition of different scaffolds are thought to have significant influence on cell behavior. Osteoblast-like cells seeded on different scaffolds have demonstrated different proliferation, adhesion, and differentiation abilities [2, 3]. Deproteinized bovine bone mineral, a xenograft with osteoconductive properties, has successfully promoted osteogenesis in different studies [4-7]. However, due to the lack of significant osteoinductive activity, it is not a suitable replacement material to autogenous bone graft, the gold standard for bone regeneration [8]. The effects of deproteinized bovine bone mineral on proliferation and differentiation of osteoblasts and osteoblast-like cells have been evaluated in a few *in vitro* experiments. However, these studies did not report comparable results [9-13].

The effect of various pharmacological agents on bone healing and their possible co-application with tissue engineering has received much attention in recent years [14, 15]. Dexamethasone (Dex) is a stimulating factor for osteoblastic proliferation, differentiation, and matrix mineralization in human pre-osteoblastic and osteoblastic cells [16, 17]. Furthermore, with a concentration range of 10^-8 ^-10^-7^ M [18], it can promote osteoblastic differentiation in mesenchymal precursors and enhance expression of the mature osteoblastic phenotype, in a time- and dose-dependent manner [19].

The objective of this *in vitro* study was to assess the effect of deproteinized bovine bone mineral on the growth and differentiation of human osteosarcoma cell line, Saos-2, and compare it with that of Dex in physiologic dose.

## Material and Methods

### Cell Culture

Human osteosarcoma cell line, Saos-2, was obtained from National Cell Bank, Iran Pasture Institute (Tehran, Iran). Saos-2 cells were cultured in a 75 cm^2^ flask containing RPMI 1640 supplemented with 10% fetal bovine serum (FBS) (Gibco, Carlsbad, California, NY, USA), and 1% Penicillin-Streptomycin (Gibco, Grand Island, NY, USA) were incubated at 37°C with 5% CO_2_/95% air atmosphere. Medium was changed every 3 days. Cells were dissociated with trypsin and were subcultured every 4-5 days at a density of 1×10^4^ cells/well in 24-well plates, in triplicate.

### Determination of Cell Proliferation

The fourth passage of cells was used for this experimental procedure. The 9 wells were divided into three groups; each group had three wells: a control group that received phosphate buffered saline (PBS), and two experimental groups in which cells were directly exposed to three to five granules of deproteinized bovine bone mineral (Bio-Oss, Geistlich, Osteohealth Biomaterials, Bern, Switzerland), or 10^-7^ M of Dex (Sigma-Aldrich, Steinheim Germany). The number of granules varied to ensure that the relative amounts of each material were similar in each group. At the indicated time intervals (24, 36, 48 hours), cells were harvested and counted using trypan blue exclusion method. The experiments were repeated three times.

### Measuring Alkaline Phosphatase Activity

Alkaline phosphatase (ALP) activity of Saos-2 cells was determined biochemically; this was used as an indicator of osteoblastic phenotype activity. Saos-2 cells were cultured in 24 wells as described previously. After 24 and 48 hours of incubation, the medium was removed and cells were extracted by adding a lysis buffer containing 0.1% (v/v) Triton X-100, 1 mM MgCl_2_, 0.1 mM ZnCl_2_ and 20 mM Tris (pH=10, Merck, Darmstadt, Germany). This procedure was performed by freezing at -70°C for 15 minutes and thawing at 37°C for 20 min, three times. For further disruption, the cultures were frozen at -80°C and stored over night. The enzyme activity was measured using paranitrophenol phosphate in the diethanolamine buffer (Merck, Darmstadt Germany) as substrate. The production of paranitrophenol by ALP was detected spectrophotometrically at 405 nm. The ALP activity results were expressed as UI/ (enzyme activity)/10 ^4^ cells.

### Statistical Analysis

The data were analyzed using Mann–Whitney U followed by Tukey-Kramer post-hoc test (SPSS 10.0, SPSS Inc., Chicago, IL, USA). The level of significance was determined at *P*<0.05.

## Results

### Cell Proliferation

**Figure 1.**
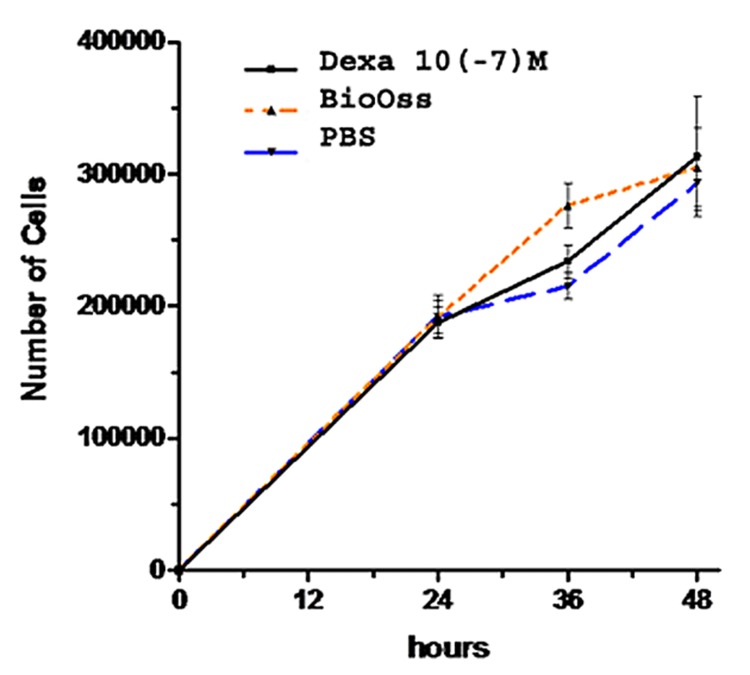
The growth curve of Saos-2 cells. The culture revealed a significant increase in the number of viable cells after 2 days in the group containing Bio-Oss

The proliferative effect of Bio-Oss and Dex on Saos-2 cells was seen after 24, 36 and 48 hours, by means of trypan blue exclusion method (Figure 1). The results from three independent experiments (*n*=3) were calculated and presented as mean±standard error (SE) (Table 1). In the first 24 hours, the rate of proliferation was comparable in the experimental and control groups. After 36 hours, the difference between Dex and control group was not yet significant (*P*>0.05) but the number of cells and the rate of growth was significantly higher in the Bio-Oss group (*P*<0.05). However, during the 36 to 48-hour period, the proliferation rate in experimental groups altered in a way that at the end of evaluation time, the number of Saos-2 cells was approximately similar in both groups.

**Table 1.** The mean±SE of cell numbers in the test and control group, counted at different time points

Hours	Control	Dexamethasone	BioOss
	**Mean±SE**	**Mean±SE**	**Mean±SE**
**0**	100000±0.00	100000±0.00	100000±0.00
**24**	188000±32.11	188000±22.89	188000±42.78
**36**	233000±12.32	252000±12.97	298000±14.38
**48**	312000±35.47	328000±31.37	409333±30.76

### Alkaline Phosphatase Activity

**Figure 2.**
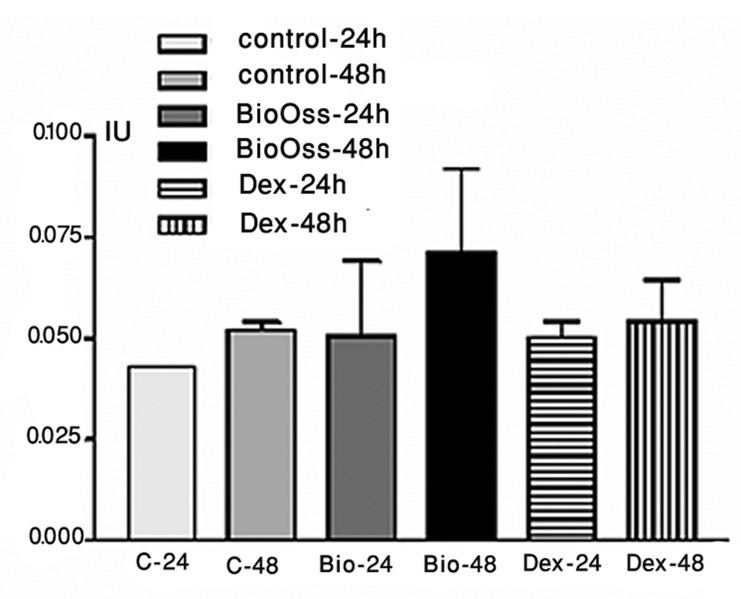
Alkaline phosphatase (ALP) activity of Saos-2 cells was determined by spectrophotometric analysis at 24 h and 48 h time points; BioOss group after 48 h revealed more activity than other groups

**Figure 3.**
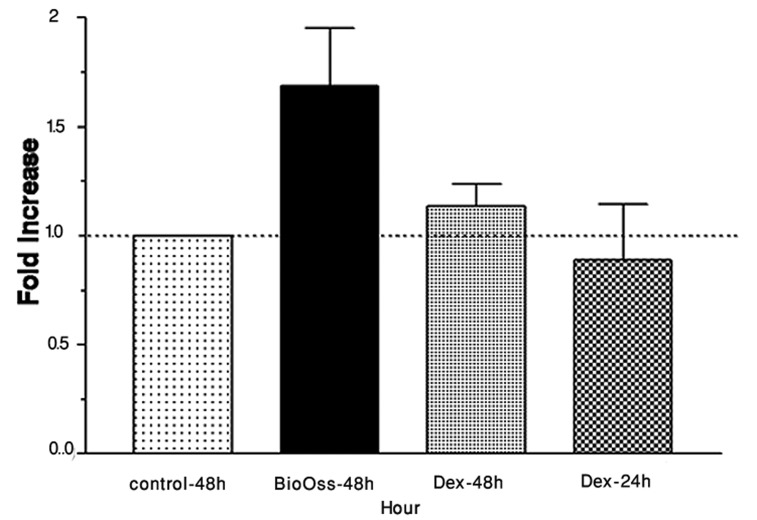
Spectrophotometry: Alkaline phosphatase (ALP) expression between Bio-Oss scaffold and Dex (10^-7^ M) revealed that ALP activity in the experimental groups was near to 1.5 times greater than control groups

The mean differences between ALP activity in control and experimental groups were analyzed after 24 and 48 hour. Both experimental groups had slightly more ALP activity than the control group, after 24 hours (Figure 2). However, after 48 hours, the phosphatase activity was significantly higher in Bio-Oss group, compared to the others (*P*<0.05) (Table 2), approximately 2 times greater than 48-hour control group (Figure 3). The difference between 48-hour Dex group and 48-hour control group was not statistically significant (*P*>0.05) (Table 2).

**Table 2.** Alkaline phosphatase activity in Saos-2 cells

Tukey's multiple comparison test	Mean Diff.	*P*-value
**C-48 vs Dex-7**	-0.1375	*P*>0.05
**C-48 vs BioOss-48**	-0.8129	*P*<0.05
**Dex-7 vs BioOss-48**	-0.6754	*P*<0.05

## Discussion

Bio-Oss is a biocompatible xenograft containing mineral component of bovine bone, with the organic portion being eliminated during particular procedures [8, 20, 21]. According to various histologic evaluations, the structural properties of Bio-Oss, such as high porosity (75% to 80%) [5, 6, 22] as well as the presence of hydroxyapatite crystals are comparable to those of human cancellous bone, provide sufficient surface area for migration and adhesion of osteogenic cells. This makes it feasible for the material to integrate with the surrounding bone [1, 8, 9, 20, 21]. 

Stephan *et al.* proved that when cultured on an organic bovine bone, osteoblastic cells can attach to the material (in a 30 to 60-min period) and proliferate [10]. Considering the short time in which attachment occurred, they also suggested that protein synthesis is not a prerequisite for cell attachment. Acil *et al.* demonstrated similar results regarding the attachment and proliferation of osteoblasts in their investigation [9]. In contrast, Petrovic *et al.* showed a decrease in proliferation of human osteoblast cells cultured on Bio-Oss, however low cell density for initial seeding was believed to be a limitation of this study [11]. The results of the present study showed that Bio-Oss improves cell proliferation as well as cell differentiation.

Various experiments have reported that the growth and activity of osteoblasts cultured on Bio-Oss can be relatively low comparing with other bone substitutes [2, 3, 11, 23, 24]. Despite these results, several clinical studies have approved this technique, and showed successful results when the material was applied as a bone substitute in implant dentistry, sinus augmentation procedures and periodontal regeneration [4-7]. Combining this xenograft with osteogenic progenitor cells [9, 25] and/or specific growth factors will compensate the lack of osteoinductivity to some extent [7].

In certain concentrations, dexamethasone is known to be an inductive factor for osteoblastic cells and increases their proliferation and differentiation [16, 17]. Moreover, it stimulates mesenchymal stem cells to differentiate into the osteogenic lineage [17, 19, 26]. Jaiswal *et al.* reported that 10^-8^ M Dex induces the highest ALP activity in human mesenchymal stem cells, whereas the rate of mineral deposition reached its maximum level at 10^-7^ M concentration [18]. This enhancing effect not only reverses in higher concentrations, but also is time- and species-dependant [19].

Jorgensen *et al.* demonstrated a significant increase in proliferation and ALP activity of human bone marrow-derived stromal cells that were treated with 10^-7^ M Dex for 7 days [17]. Eijken *et al.* used a human pre-osteoblast model to assess the role of 10^-7^ M Dex during a 3 week period. They showed a significant increase in cellular proliferation and ALP activity. They also suggested that to effectively exploit the beneficial effects of Dex, its application during the early developmental stages is necessary [16].

In a recent study, Song *et al.* successfully induced ectopic bone formation by pretreatment of human mesenchymal stem cells with 10^-8^ M Dex [27]. The required treatment duration with Dex for attaining full osteogenic differentiation was determined to be over 3 weeks. Guzman-Morales and co-workers also confirmed previous studies’ results and introduced Dex (10^-7^ M or 10^-8^ M) as an inducing factor for human bone marrow mesenchymal stem cells, though with a collateral inhibitory effect on cell proliferation [28]. One should notice that not all investigations have come up with a unanimous conclusion and the effect of Dex on osteoblastic proliferation and differentiation still remains controversial [29]. The results of the present study indicated that 10^-7^ M Dex slightly increased the proliferation rate and the induced ALP activity was also lower than that in the Bio-Oss group. While in most supporting studies, the enhancing effect of Dex on cellular proliferation has been evaluated in longer periods (weeks) [16, 17], our results indicate that although, at the concentration of 10^-7^ M, Dex is less effective than Bio-Oss in enhancing cellular proliferation, its effects start within hours after addition to the culture.

Recently, some experiments have focused on the addition of Dex to sustained releasing scaffolds, in order to add an osteoinductive effect. In an *in vitro* study, Kim *et al.* loaded PLGA scaffolds with ascorbate-2-phosphate and Dex, so that a continuous release of these substances could be obtained. The results showed significant calcium deposition on the experimental cultures [26]. The subsequent *in vivo* study also contributed to successful outcomes in osteogenesis [30-33]. Although the current results do not demonstrate the enhancing effect of Dex to be more than that of Bio-Oss, further *in vitro *and *in vivo* studies need to be performed to determine the feasibility and the efficacy of co-application of Dex and Bio-Oss, as a novel method in tissue engineering.

## Conclusion

According to the results of this *in vitro *study, in a 48 hours evaluation period on Saos-2 cells, both Bio-Oss and 10^-7^ M Dex increased the number of cells and the ALP activity. However, the obtained increase in Dex-treated cells was not statistically significant.
